# Viral load assay performs comparably to early infant diagnosis assay to diagnose infants with HIV in Mozambique: a prospective observational study

**DOI:** 10.1002/jia2.25422

**Published:** 2020-01-08

**Authors:** Adolfo Vubil, Carina Nhachigule, Osvaldo Loquiha, Bindiya Meggi, Nedio Mabunda, Timothy Bollinger, Jilian A Sacks, Ilesh Jani, Lara Vojnov

**Affiliations:** ^1^ Instituto Nacional de Saude Maputo Mozambique; ^2^ Clinton Health Access Initiative Maputo Mozambique; ^3^ Department of Mathematics and Informatics Universidade Eduardo Mondlane Maputo Mozambique; ^4^ World Health Organization Geneva Switzerland

**Keywords:** diagnostics, paediatrics, testing, virology, LMIC, Africa, viral load monitoring

## Abstract

**Introduction:**

Viral load testing is essential to manage HIV disease, especially in infants and children. Early infant diagnosis is performed using nucleic‐acid testing in children under 18 months. Resource‐limited health systems face severe challenges to scale‐up both viral load and early infant diagnosis to unprecedented levels. Streamlining laboratory systems would be beneficial to improve access to quality testing and to increase efficiency of antiretroviral treatment programmes. We evaluated the performance of viral load testing to serve as an early infant diagnosis assay in children younger than 18 months.

**Methods:**

This study was an observational, prospective study, including children between one and 18 months of age who were born to HIV‐positive mothers in 134 health facilities in Maputo City and Maputo Province, Mozambique. Dried blood spot specimens from heel or toe pricks were collected between January and April 2018, processed using SPEX buffer for both assays, and tested for routine EID and VL testing using the Roche CAP/CTM HIV‐1 Qualitative v2 and Roche CAP/CTM HIV‐1 Quantitative v2 assays respectively. The sensitivity, specificity and positive and negative predictive values were estimated using the EID results as the reference standard.

**Results:**

A total of 1021 infants were included in the study, of which 47% were female. Over 95% of mothers and children were on antiretroviral treatment or received antiretroviral prophylaxis respectively. The sensitivity and specificity of using the viral load assay to detect infection were 100% (95% CI: 96.2 to 100%) and 99.9% (95% CI: 99.4 to 100%). The positive and negative predictive values were 99.0% (95% CI: 94.3 to 100%) and 100% (95% CI: 99.6 to 100%). The McNemar's test was 1.000 and Cohen's kappa was 0.994.

**Conclusions:**

The comparable performance suggests that viral load assays can be used as an infant diagnostic assay. Infants with either low levels of viraemia or high cycle threshold values should be repeat tested to ensure the result is truly positive prior to treatment initiation, regardless of assay used. Viral load assays could replace traditional early infant diagnosis testing, substantially streamlining molecular laboratory services for children and lowering costs, with the additional advantage of providing baseline viral load results for antiretroviral treatment management.

## 1 Introduction

Although significant increases in early infant diagnosis (EID) testing coverage has occurred globally in the past ten years, only approximately 50% of HIV‐exposed infants received an EID test within the first two months of life in 2017 1. In Mozambique, of the estimated nearly 130,000 HIV‐exposed infants, approximately 63% received an EID test in 2018. Although about 75% of HIV‐positive pregnant women are accessing treatment in Mozambique, mother‐to‐child transmission rates remain relatively high at 11% 1, 2.

HIV‐positive infants are particularly vulnerable. HIV‐related mortality peaks at two to three months of age for untreated infants infected *in utero* 3. Furthermore, it was estimated that approximately 50% of HIV‐positive infants may die before two years of life if left untreated 4. Unfortunately, however, globally only approximately 50% of HIV‐positive infants were initiated on antiretroviral treatment in 2017 1. Efficient and effective testing systems are critical to ensure infants are identified early and quickly linked to life‐saving ART.

Challenges remain in the EID testing network, both globally and within Mozambique. Reagent stock outs, small infant testing volumes, requirements for sample batching and high commodity prices persist 5. Additionally, the logistics of managing two different yet similar assays for EID and viral load testing may fragment supply chain management and procurement. Many of these challenges, alone or in combination, can lead to testing delays or stoppages. Furthermore, test suppliers have noted manufacturing challenges and delays due to the small EID market size and sporadic procurement. Due to the high morbidity and mortality rates of HIV‐infected infants, delays in diagnosis can be detrimental. HIV molecular diagnostic laboratories in Mozambique typically conduct both EID and viral load testing in the same laboratory and using the same technologies. However, performing different tests in the same laboratory and on the same equipment can cause complications; therefore, separation of sample processing and testing as distinct workflow and processing stations are often implemented.

In 2010, the World Health Organization recommended that several assays could be used to diagnose infants and children under 18 months of age, including HIV RNA testing on plasma or DBS 6. Consolidating and simplifying testing using viral load assays as an infant diagnostic may reap significant benefits, particularly as countries could access often lower viral load prices, remove the necessity to batch samples, unify procurement and consolidate volumes to ensure consistent reagent supply. We, therefore, conducted a diagnostic accuracy study to determine the performance of a viral load assay to accurately diagnose HIV infection in HIV‐exposed infants under 18 months of age in Mozambique.

## 2 Methods

### 2.1 Study design and participants

This was an observational, prospective study that included infants between one and 18 months of age born to HIV‐1‐positive mothers in need of a routine HIV Early Infant Diagnosis (EID) test. Infants excluded from the study were those less than one month and older than 18 months of age or with low quality specimen, according to the rejection criteria used for HIV EID routine testing in Mozambique. Samples were collected from 134 health facilities that attend to infants born to HIV‐positive mothers in Maputo City and Maputo Province between January and April 2018. Dried blood spot specimens were collected and referred to the National Institute of Health Reference laboratory in Maputo for EID routine testing and viral load testing. Demographic and clinical data for study participants were collected using a routine EID form, including gender, age and exposure to maternal treatment and infant prophylaxis. Because patient identifiers were not collected, remnant spots from routine clinical samples were used, and only standard clinical test results were provided to caregivers and clinicians, individual consent was waived and approval by the Institutional Review Boards that reviewed the protocol.

### 2.2 Test methods

Dried blood spot specimens (Whatman 903, GE Healthcare Biosciences, Pittsburgh, PA, USA) were drawn from the heel or toe pricks of eligible infants and transported within three weeks to the reference laboratory for early infant diagnosis HIV‐1 PCR testing using the Roche CAP/CTM 96 HIV‐1 Qualitative Test v2 (Roche Molecular Diagnostics, Branchburg, NJ, USA). Low quality specimens were excluded, including those without full dried blood spots and when two or more cards were in the same ziplock bag without glassine paper between the cards. This test detects extracellular and intracellular HIV‐1 RNA and proviral DNA in whole blood specimens. The Roche software automatically corrects for the haematocrit value in dried blood spot specimens.

Infants were determined positive when the Roche CAP/CTM qualitative EID assay reported a detectable result with a cycle threshold less than 31. National policy states that laboratories should implement an indeterminate range that includes results with a cycle threshold (Ct) value of 31 or greater using the Roche CAP/CTM EID assay; infants with an initial EID cycle threshold value in this range received a second EID test, if possible, either on the same or a new sample before a definitive test result is determined. The EID definitive result was determined based on the test result of the second (repeat) Roche CAP/CTM EID test. If the repeat EID test result was target not detected, the infant was determined to be HIV negative. If the repeat EID test result was a detectable result, the infant was determined to be HIV positive. In this study, the viral load results were not used to determine positivity nor were they returned to the healthcare facility or caregiver.

After the EID test result, HIV‐1 viral load testing was performed using the Roche CAP/CTM 96 HIV‐1 Quantitative Test v2 (Roche Molecular Diagnostics, Branchburg, NJ, USA) using remnant specimens. The Sample Pre‐Extraction (SPEX) solution was used for DBS elution for both qualitative and quantitative testing. The routine EID test results were returned to the healthcare facility and caregiver per national guidelines. Viral load test results were not provided to health care facilities or caregivers and were used for study purposes only.

The reference laboratory routinely participated in and passed external quality assessment programmes for both EID and viral load (provided by the Center for Disease Control and Prevention, Atlanta, USA) prior to and during the study period.

### 2.3 Analysis

The sensitivity and specificity of quantitative testing (HIV viral load) as well as the positive and negative predictive values were estimated using the qualitative assay (EID) as the reference. Cohen's kappa and McNemar's test were also performed. Only valid results (detectable or undetectable) were used for these calculations; invalid results were excluded. Additionally, we conducted a sub‐analysis to determine the diagnostic accuracy of the viral load and EID assays if the indeterminate range is not implemented and the EID definitive result is based solely on the initial EID test result. We compared demographic data between HIV‐positive and HIV‐negative groups using the Chi‐square test. Data were analysed using SAS/STAT software version 9.4 (SAS Institute Inc, Cary, NC, USA).

### 2.4 Protocol approval

This study was approved by the Mozambique National Health Bioethics Committee, Advarra Institutional Review Board in the USA, and the Ethics Review Committee from the World Health Organization, Geneva, Switzerland.

## 3 Results

### 3.1 Patient population

**Figure 1:**
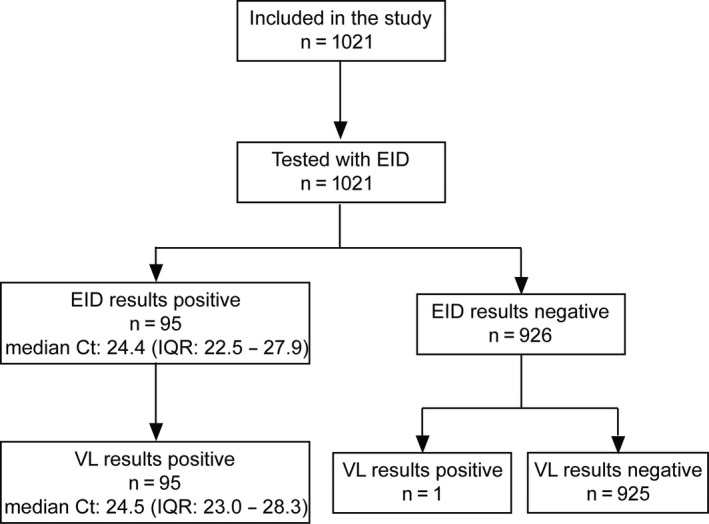
Flow diagram of study participants.

A total of 1021 infants were included in the study (Figure 1), of which 46.9% were female (Table 1). The median age of all infants was 33 days (IQR: 31 to 61 days), while 68.9% were less than 60 days old. The proportion of infants who tested positive for HIV with the EID assay was 9.3% (n = 95). Nearly all mothers were on antiretroviral treatment (95.8%) and 95.7% of infants were given antiretroviral prophylaxis at the time of testing. The majority (87.7%) of infants were exclusively breastfed.

**Table 1:** Study participant characteristics

	EID test result	
	Negative	Positive	Total	Chi‐square	t‐test
	n (%)	n (%)	n (%)	*p*‐value	*p*‐value
Sex					
Female	428 (46.2%)	51 (53.7%)	479 (46.9%)	0.276	
Male	417 (45.0%)	39 (41.1%)	456 (44.7%)		
Not available	81 (8.7%)	5 (5.3%)	86 (8.4%)		
Age					
30 to 60 days	660 (71.3%)	43 (45.3%)	703 (68.9%)		
61 to 90 days	62 (6.7%)	11 (11.6%)	73 (7.1%)		
91 to 180 days	51 (5.5%)	16 (16.8%)	67 (6.6%)	<0.001	
181 to 270 days	51 (5.5%)	10 (10.5%)	61 (6.0%)		
≥271 days	28 (3.0%)	8 (8.4%)	36 (3.5%)		
Not available	74 (8.0%)	7 (7.4%)	81 (7.9%)		
Median age (IQR)	33 (31 to 60)	64.5 (43 to 162)	33 (31 to 61)		<0.001
Infant prophylaxis					
None	12 (1.3%)	4 (4.2%)	16 (1.6%)		
AZT/NVP	888 (95.9%)	89 (93.7%)	977 (95.7%)	0.055	0.312
Not available	26 (2.8%)	2 (2.1%)	28 (2.7%)		
Mothers prophylaxis					
None	7 (0.8%)	5 (5.3%)	12 (1.2%)	0.003	0.033
ART/NVP/AZT + 3TC	893 (96.4%)	85 (89.5%)	978 (95.8%)		
Not available	26 (2.8%)	5 (5.3%)	31 (3.0%)		
Breastfeeding					
None	49 (5.3%)	8 (8.4%)	57 (5.6%)	0.132	
Exclusive	820 (88.6%)	75 (78.9%)	895 (87.7%)		
Mixed	9 (1.0%)	2 (2.1%)	11 (1.1%)		
Not available	48 (5.2%)	10 (10.5%)	58 (5.7%)		
Total	926 (90.7%)	95 (9.3%)	1021		

### 3.2 Data analysis

Of the 95 infants determined to be HIV positive, the median viral load on the quantitative assay was 944,421 copies/mL (IQR: 51,715 to 2,405,127), while the median cycle threshold value on the qualitative assay was 24.4 (IQR: 22.5 to 27.9). The median cycle threshold value on the quantitative assay was 24.5 (IQR: 23.0 to 28.3). The majority (90 of 95) quantitative results were within four cycles of the qualitative results (−1 to + 3 cycles) (Table S1). Just over half (55%, n = 53) of the HIV‐positive infants had an EID cycle threshold value less than 25, 30% (n = 29) had a cycle threshold value between 25 and 30, 14% (n = 13) had a cycle threshold value between 30 and 35, and 1% (n = 1) had a cycle threshold value greater than 35.

The HIV‐positive infants were significantly older than the HIV negative (64.5 vs. 33 days, *p* < 0.001), while 71.3% of HIV‐negative infants were less than 60 days old only 45.3% of HIV‐positive infants were less than 60 days old (*p* < 0.001). Of the HIV‐positive infants, 89.5% of mothers were on treatment compared to 96.5% of HIV‐negative infants (*p* = 0.033), while 93.7% of HIV‐positive infants were on prophylaxis compared to 95.9% of HIV‐negative infants (*p* = 0.312). Ninety‐five percent of HIV‐positive infants were exposed to antiretroviral drugs through either maternal treatment or infant prophylaxis, while 99% of HIV‐negative infants were similarly exposed.

The sensitivity and specificity of the viral load assay to correctly diagnose HIV infection compared to the EID assay was 100.0% (95% CI: 96.2 to 100%) and 99.9% (95% CI: 99.4 to 100%) respectively (Table 2). The positive and negative predictive values were 99.0% (95% CI: 94.3 to 100%) and 100.0% (95% CI: 99.6 to 100%). Cohen's kappa was 0.994 (95% CI: 0.989 to 0.999) and McNemar's test was 1.000 (*p* = 0.3173). One infant was falsely positive by the viral load assay. The infant was 84 days old at the time of testing, receiving infant prophylaxis, and exposed to maternal antiretroviral treatment. The viral load result was < 400 copies/mL and Ct value of 35.3 (Patient #5, Table 3). The infant had an initial positive EID test results with a Ct value of 31.6. The subsequent test on the same sample was negative and, therefore, the definitive result was HIV negative.

**Table 2:** Results of viral load quantitative testing compared with early infant diagnosis qualitative testing

		Viral load result					
		Detected	Not detected	Sensitivity (95% CI)	Specificity (95% CI)	PPV (95% CI)	NPV (95% CI)
EID result	Positive	95	0	100.0%	99.9%	99.0%	100.0%
	Negative	1	925	(96.2 to 100.0%)	(99.4 to 100.0%)	(94.3 to 100%)	(99.6 to 100%)

Cohen's kappa (95% CI): 0.994 (0.989 t 0.999). McNemar's test (*p*): 1.000 *(p* = 0.3173).

**Table 3:** Results of viral load quantitative testing compared with early infant diagnosis qualitative testing, without utilizing an indeterminate range

		Viral load result					
		Detected	Not detected	Sensitivity (95% CI)	Specificity (95% CI)	PPV (95% CI)	NPV (95% CI)
EID result	Positive	95	4	96.0%	99.9%	99.0%	99.6%
	Negative	1	925	(90.0 to 98.9%)	(99.4 to 100.0%)	(94.3 to 100%)	(98.9 to 99.9%)

Cohen's kappa (95% CI): 0.972 (0.961 to 0.982). McNemar's test (*p*): 1.800 (*p* = 0.1797).

Mozambique implements an indeterminate range that includes results with a cycle threshold on the Roche CAP/CTM EID assay of 31 or greater. Infants with an initial EID cycle threshold result equal to or above 31 receive a second EID test before a diagnosis is determined. We, therefore, sought to determine the performance of the EID and viral load assays if the indeterminate range was not applied. Without considering the indeterminate range, the sensitivity of the viral load assay was 96.0% (95% CI: 90 to 98.9%), while the specificity was 99.9% (95% CI: 99.4 to 100%) (Table 3). In this sub‐analysis, there were five potentially false‐positive viral load test results. The initial EID cycle threshold results of each were 40.9, 34.3, 34.2, 33.5 and 31.6 (Table 4). However, when the indeterminate range was implemented and follow‐up testing conducted, four of the five initially EID positives tested negative (target not detected) on either the same or a new sample and were determined to be HIV negative. Without an indeterminate range, the false‐positive rate of the EID assay was 4.0% (95% CI: 1.1 to 10.0).

**Table 4:** Initial and follow‐up early infant diagnosis and viral load reults of all samples with initial early infant diagnosis qualitative cycle threshold values above 31

	First sample		Second sample				
Patient	Ct 1	Ct 2	Ct 1	Ct 2	EID definitive result	Viral load result (copies/mL)	Viral load Ct
1	40.9	Not detected			Negative	Not detected	N/A
2	34.3	Not detected	Second DBS not collected		Negative	Not detected	N/A
3	33.5	Not detected			Negative	Not detected	N/A
4	34.2	Not tested	Not detected	N/A	Negative	Not detected	N/A
5	31.6	Not detected			Negative	<400	35.3
6	31.6	Not tested	29.6	N/A	Positive	1453	32.9
7	31.6	32.2	32.0	N/A	Positive	1204	34.2
8	32.8	Not tested			Positive	<400	38.2
9	31.3	31.1			Positive	577	NA
10	31.5	31.2	Second DBS not collected		Positive	<400	NA
11	31.2	31.9			Positive	2140	34.6
12	32.4	Not tested			Positive	<400	NA
13	33.2	Not tested	Positive	<400	NA		
14	31.5	Not tested	Positive	506	NA		

Grey shading, false positive case; NA, not available; N/A, not applicable or additional testing unnecessary as definite result determined; Not detected, target not detected; Not testing, additional sample unavailable.

## 4 Discussion

Previous studies have suggested that viral load testing can be used as an infant diagnostic 7, 8, 9; however, most studies were conducted in developed settings and prior to 2005 when maternal treatment and infant prophylaxis were poorly implemented and not yet widespread in sub‐Saharan Africa. Exposure to antiretroviral drugs can significantly reduce mother to child transmission 10, 11, 12, 13, 14, but also reduce the amount of virus at diagnosis of HIV‐infected infants 15. The present study is the first to find similarly high sensitivity (100%) and specificity (99.9%) of the viral load assay when being used as a diagnostic for early infant diagnosis in a high HIV burden setting with high exposure rates to antiretroviral drugs. In 2010, the World Health Organization recommended that early infant diagnosis can be performed targeting a variety of nucleic acids, including DNA and RNA 6; however, it is not clear that the guidelines recommend the use of either qualitative or quantitative assays. The data presented here provide a proof of principle that both assay types can be used to accurately diagnose HIV‐exposed infants.

The results of this study are highly generalizable. Sample sizes within the highest cycle threshold or lower viral load values were included with nearly 30% of infants having an initial EID cycle threshold result above 30. No infants less than one month of age were included, so the generalizability to birth testing may be limited. While HIV‐positive infants tested at birth generally have lower levels of virus 15, we still observed high sensitivity amongst infants with high Ct values or low levels of virus, regardless as to age. Furthermore, sampling was done consecutively until the sample size was reached representing a typical population attending primary health care facilities in Mozambique. Finally, maternal treatment and infant prophylaxis rates were very high in the included population: greater than 95% of mothers and infants were on treatment or receiving prophylaxis respectively.

Previous studies have reported discordant or false‐positive values using molecular technologies, either qualitative or quantitative assays 16, 17, 18. While there is a correlation between the cycle threshold value and the level of virus in a sample, the various circulating viral subtypes, assay techniques, and use of older technologies can led to discordant PCR results. Unfortunately, no assay is or will likely be perfect. However, it is understood that the viral load in infants can predict the Ct values of qualitative assays 19. Due to the changing dynamics of mother‐to‐child transmission – increased proportions of women in PMTCT programs, incident infections and later MTCT transmission, lower levels of virus at infant diagnosis due to exposure to maternal treatment and infant prophylaxis, and introduction and widespread use of dolutegravir in pregnant and breastfeeding women – definitive HIV diagnosis of infants will become more challenging, yet previous studies and our results suggest that this may not be due to technological or performance barriers.

WHO strongly recommended implementing an indeterminate range to improve the accuracy of nucleic acid‐based early infant diagnosis assays in 2018 20. In line with global guidelines, Mozambique has been implementing an indeterminate range, when interpreting early infant diagnosis test results. Even though detectable, all infants with an EID result equal to or above a cycle threshold of 31 on the Roche CAP/CTM HIV‐1 qualitative v2 technology require additional testing, preferably on the same sample, prior to return of the result to the facility. Mozambique began implementing an indeterminate range prior to the 2018 WHO guidelines and thus had determined that an indeterminate range of 31 using the Roche CAP/CTM EID assay was preferable in their setting. Furthermore, it is important to note that the Ct values of an indeterminate range will vary across different assays. Interestingly, if we ignored the indeterminate range and definitive diagnosis, thus relying solely on whether the first EID test was detectable, the false‐positive rate of the EID assay was 4.0%. There were four false‐positive results using the initial EID test, all of which were negative using the viral load assay. Relying on the detectability and manufacturer result interpretation of a single EID test would have resulted in these infants being considered HIV‐positive and potentially put on lifelong treatment unnecessarily. Although confirmatory testing is recommended globally and in nearly all countries, implementation has been poor and thus cannot be relied on entirely to prevent false‐positive infants being put on treatment unnecessarily 6, 21. These data reiterate the importance of implementing an indeterminate range for test results with low levels of viraemia, particularly in countries with high antiretroviral drug exposure and decreasing mother to child transmission rates, in order to reduce the proportion of false positives and potential unnecessary lifelong treatment 20, 21.

Significant challenges and barriers to increasing timely access to early infant diagnosis continue to stall the success of EID systems, including lack of price parity with viral load assays, reagent stock outs, duplicative workflows and sample batching. Utilizing viral load as an infant diagnostic test may support a more efficient laboratory system. Reagents would be purchased at the often lower viral load price, thus leveraging the significant viral load volumes and associated negotiation power. Stock outs would be reduced as viral load reagents are often better managed due to the significant and consistent volumes and utilization. Furthermore, separate and complicated workflows in the laboratory would be eliminated as early infant diagnosis and viral load samples can be tested together and systems integrated. This would also reduce the need to wait for a full batch of early infant diagnosis samples prior to testing – early infant diagnosis samples could be run immediately after processing with a batch of samples for viral load testing. Furthermore, using viral load as an infant diagnostic would also provide the laboratory and clinician with a baseline viral load test result, if desired. Finally, though contamination may be possible across samples, particularly during sample preparation, the most common molecular technologies are closed systems that significantly limit the possibility of cross‐sample contamination during nucleic acid extraction and amplification.

In order to support implementation of using viral load as an infant diagnostic, manufacturers would ideally seek regulatory approval within their current and/or future viral load assays' intended use claims. In fact, dual claims have been sought by suppliers already 21. Using viral load as an infant diagnostic would simplify supplier manufacturing since only viral load reagents would be needed and allow suppliers to focus on producing and supporting this primary, high volume product. Additionally, there are now point‐of‐care and near point‐of‐care early infant diagnosis as well as viral load assays that could be considered for dual claim regulatory approval processes and implementation.

There were several limitations in this study. This study was not powered to conduct sub‐analyses based on treatment exposure or time at testing. Ideally similar studies for additional technologies would be conducted; however, the present study provides a clear proof of principle and confirmation that viral load can be used as an infant diagnostic within current programmatic settings in high HIV burden countries. Policy adoption and early implementation can be considered to maximize resources, streamline laboratory systems and provide greater access to testing. When used to monitor patients on treatment, plasma is the preferred sample type for viral load testing 22; however, dried blood spots are the preferred sample type for early infant diagnosis in Mozambique and many other high burden countries. This study did not review the performance of other sample types, including plasma, and it should be noted that the dried blood spot samples for viral load testing were processed similarly to those for EID (i.e. using SPEX buffer) before extraction and amplification using the viral load assay. Repeat testing was unfortunately not available for all indeterminate cases either on the same specimen or a new specimen (n = 4). This may have been due to one or several programmatic challenges and highlights the real world nature of this study. Although all were detectable initially, they had a Ct value greater 31. These cases were reported as positive based on that first test and, though the viral load result was not incorporated within the diagnostic interpretation, all had a detectable viral load.

Interestingly, the age at testing was significantly different between the HIV‐positive and HIV‐negative infants. HIV‐positive infants were nearly twice as old as the HIV‐negative infants (64.5 vs. 33 days) at the time of sample collection. Similar data were observed previously 23, 24. No test requisition forms indicated that the specimen was a later testing timepoint in the early infant diagnosis algorithm. Presumably, this may have been in part due to late mother‐child presentation to the health care facility, perhaps due to stigma and discrimination issues, healthcare worker attitudes, or weak case‐finding strategies, and may need further study. Furthermore, late presentation may also be correlated with other characteristics that hinder service utilization and/or retention of mothers and infants, such as young maternal age and status disclosure 25, 26. Additionally, though nearly 90% of mothers of HIV‐positive infants were on treatment, the proportion was significantly lower than the mothers of HIV‐negative infants (96.5%). The high rate of HIV‐positive infants from women on ART was surprising and suggests the need for better adherence support, earlier identification and initiation of ART, and more effective ART regimens for HIV‐positive pregnant women. No differences were observed between the groups with regards to infant prophylaxis, perhaps indicating that many of the HIV‐positive infants were infected *in utero* before birth and initiation of infant prophylaxis. Expansion and improvements in prevention of mother to child transmission services and ensuring mothers are closely adherent to treatment throughout pregnancy and breastfeeding are critical.

## 5 Conclusions

The results in this study highlight that viral load quantitative assays can be used as an infant diagnostic test. This may allow for streamlined manufacturing as well as simplified laboratory logistics and procurement, while also providing additional clinical information. However, it is imperative that manufacturers submit the appropriate change notification to regulatory bodies in order to fully support countries considering implementation. As early infant diagnosis coverage rates have remained stagnant over recent years, tried and novel case‐finding interventions coupled with the intervention presented here may be necessary to improve access to this essential test as it remains a gateway to identification of HIV‐infected infants in need of life‐saving treatment.

## Competing interest

The authors declare no competing interest.

## Authors' contributions

AV designed study, oversaw testing, collated data, interpreted results, substantially commented on the manuscript drafts and approved the finalized version of the manuscript. CN, BM, and NM conducted testing, collated data, interpreted results, substantially commented on the manuscript drafts and approved the finalized version of the manuscript. TB and JA: interpreted results, substantially commented on the manuscript drafts and approved the finalized version of the manuscript. OL conducted data analysis, interpreted results, substantially commented on the manuscript drafts and approved the finalized version of the manuscript. IJ and LV: designed study, interpreted results, wrote the first draft and produced the finalized version of the manuscript.

## Supporting information


**Table S1. **Cycle threshold values for the qualitative (EID) and quantitative (VL) tests of each HIV‐positive infant
