# ERRATA

**DOI:** 10.1002/jia2.25465

**Published:** 2020-03-23

In the article 1, the following errors were published on e25422.

1. In Table 4, there were errors in format that caused confusion in content. In Patients 13 and 14, the last three values in these rows were shifted to the wrong columns. These should be corrected to improve clarity.

The correct Table 4 should be:

**Table 4:** Initial and follow‐up early infant diagnosis and viral load results of all samples with initial early infant diagnosis qualitative cycle threshold values above 31

Patient	First sample		Second sample		EID definitive result	Viral load result (copies/mL)	Viral load Ct
	Ct 1	Ct 2	Ct 1	Ct 2			
1	40.9	Not detected	Second DBS not collected		Negative	Not detected	N/A
2	34.3	Not detected			Negative	Not detected	N/A
3	33.5	Not detected			Negative	Not detected	N/A
4	34.2	Not tested	Not detected	N/A	Negative	Not detected	N/A
5	31.6	Not detected			Negative	<400	35.3
6	31.6	Not tested	29.6	N/A	Positive	1,453	32.9
7	31.6	32.2	32.0	N/A	Positive	1,204	34.2
8	32.8	Not tested	Second DBS not collected		Positive	< 400	38.2
9	31.3	31.1			Positive	577	NA
10	31.5	31.2			Positive	<400	NA
11	31.2	31.9			Positive	2140	34.6
12	32.4	Not tested			Positive	<400	NA
13	33.2	Not tested			Positive	<400	NA
14	31.5	Not tested			Positive	506	NA

Grey shading, false positive case, N/A, not applicable or additional testing unnecessary as definite result determined; NA, not available Not detected, target not detected; Not testing, additional sample unavailable.

- In 3.2 Data analysis, last paragraph, the text was inconsistent with the data on Table 4.

The sentences read:

In this sub‐analysis, there were five potentially false‐positive viral load test results. The initial EID cycle threshold results of each were 40.9, 34.3, 34.2, 33.5 and 31.6 (Table 4). However, when the indeterminate range was implemented and follow‐up testing conducted, four of the five initially EID positives tested negative (target not detected) on either the same or a new sample and were determined to be HIV negative.

The sentences should have read:

In this sub‐analysis, there were five potentially false‐negative viral load test results. The initial EID cycle threshold results of each were 40.9, 34.3, 34.2, 33.5 and 31.6 (Table 4). However, when the indeterminate range was implemented and follow‐up testing conducted, all five initially EID positives tested negative (target not detected) on either the same or a new sample and were determined to be HIV negative. There was not enough sample to retest the viral load results from the fifth patient in particular.

- The format of some abbreviations was incorrect and has been fixed throughout the article to improve clarity.

Antiretroviral treatment (ART)

Early infant diagnosis (EID)

Mother‐to‐child transmission (MTCT)

Prevention of mother‐to‐child transmission (PMTCT)

Viral load (VL)

World Health Organization (WHO)
