# Supplementary material for: Viral load assay performs comparably to early infant diagnosis assay to diagnose infants with HIV in Mozambique: a prospective observational study
Source: J Int AIDS Soc. 2020 Jan 8;23(1):e25422. doi: 10.1002/jia2.25422 (PMC6948022; doi:10.1002/jia2.25422)
Supplement: Supplementary file 1 — Table S1. Cycle threshold values for the qualitative (EID) and quantitative (VL) tests of each HIV‐positive infant [file JIA2-23-e25422-s001.docx]

Supplementary Table 1. Cycle threshold values for the qualitative (EID) and quantitative (VL) tests of each HIV-positive infant.

| **Patient** | **EID Ct value** | **VL Ct value** |
| --- | --- | --- |
| **1** | 27.9 | 27.2 |
| **2** | 19.2 | 19.2 |
| **3** | 22.9 | 23.4 |
| **4** | 24.4 | 25.9 |
| **5** | 23.9 | 24.5 |
| **6** | 19.4 | 18.8 |
| **7** | 21 | 21 |
| **8** | 27.6 | 27.2 |
| **9** | 28.6 | 29 |
| **10** | 25.1 | 24.4 |
| **11** | 24.5 | 24.8 |
| **12** | 23.1 | 23.1 |
| **13** | 21.6 | 22.1 |
| **14** | 20.5 | 21.4 |
| **15** | 23.1 | 22.5 |
| **16** | 24.3 | 24.9 |
| **17** | 23.7 | 23.4 |
| **18** | 23.1 | 22.9 |
| **19** | 28.2 | 33.2 |
| **20** | 21.8 | 22 |
| **21** | 23.4 | 23.1 |
| **22** | 22.5 | 22.1 |
| **23** | 21.6 | 21.8 |
| **24** | 22 | 22.2 |
| **25** | 24.9 | 25.2 |
| **26** | 26.3 | 27.5 |
| **27** | 22.6 | 23.3 |
| **28** | 26.5 | 29.2 |
| **29** | 23.3 | 23.8 |
| **30** | 23 | 23.9 |
| **31** | 20 | 19.8 |
| **32** | 25.7 | 26.4 |
| **33** | 23.9 | 23.9 |
| **34** | 23.3 | 23.6 |
| **35** | 22.1 | 27.4 |
| **36** | 32.8 | 38.2 |
| **37** | 28.1 | 29.6 |
| **38** | 22.6 | 23.9 |
| **39** | 29.3 | 30.3 |
| **40** | 21.8 | 21.6 |
| **41** | 29 | 29.2 |
| **42** | 25.3 | 25.6 |
| **43** | 21.4 | 21.5 |
| **44** | 29.3 | 31.2 |
| **45** | 21.8 | 21.7 |
| **46** | 21.6 | 22.4 |
| **47** | 25 | 27.6 |
| **48** | 26.9 | 26 |
| **49** | 26.8 | 28.5 |
| **50** | 21.4 | 22 |
| **51** | 29.6 | 30.7 |
| **52** | 21.9 | 23 |
| **53** | 22.6 | 23.9 |
| **54** | 21.1 | 22.5 |
| **55** | 26 | 28.2 |
| **56** | 25.2 | 26.2 |
| **57** | 25.2 | 26.4 |
| **58** | 22.8 | 23.5 |
| **59** | 28.5 | 29.3 |
| **60** | 20.8 | 21.4 |
| **61** | 26.7 | 28.3 |
| **62** | 31.3 | NA |
| **63** | 30.5 | NA |
| **64** | 31.5 | NA |
| **65** | 32.4 | NA |
| **66** | 31.6 | 32.9 |
| **67** | 33.2 | NA |
| **68** | 31.5 | NA |
| **69** | 31.6 | 34.2 |
| **70** | 31.6 | 35.3 |
| **71** | 21.2 | 23.5 |
| **72** | 28.9 | 29.5 |
| **73** | 29.8 | 31.5 |
| **74** | 26.3 | 28.1 |
| **75** | 25.9 | 28.7 |
| **76** | 26.1 | 27.3 |
| **77** | 22.5 | 22.9 |
| **78** | 28.3 | 28.3 |
| **79** | 30.3 | 31.3 |
| **80** | 23.5 | 25.3 |
| **81** | 23.3 | 23.8 |
| **82** | 27.2 | 28.5 |
| **83** | 24.3 | 26.1 |
| **84** | 21.5 | 23.6 |
| **85** | 22.4 | 24.1 |
| **86** | 18.4 | 19.4 |
| **87** | 25.9 | 28.2 |
| **88** | 22.3 | NA |
| **89** | 23.3 | NA |
| **90** | 24.8 | 24.5 |
| **91** | 24.1 | 23.8 |
| **92** | 25.1 | 24.5 |
| **93** | 23.4 | 24.5 |
| **94** | 31.2 | 34.6 |
| **95** | 24.8 | 24.8 |

NA: not available
